# Device Structure, Light Source Height, and Sunset Time Affect the Light-Trap Catching of Tea Leafhoppers

**DOI:** 10.3390/plants13020241

**Published:** 2024-01-15

**Authors:** Lei Bian, Huihua Ji, Xiaoming Cai, Guo Cheng, Xiaoqun Xie, Xiaofeng Duan, Zongmao Chen

**Affiliations:** 1 Key Laboratory of Biology, Genetics, and Breeding of Special Economic Animals and Plants, Ministry of Agriculture and Rural Affairs, Tea Research Institute, Chinese Academy of Agricultural Science, 9 Meiling South Road, Xihu District, Hangzhou 310008, China; 2 College of Optics and Electronic Technology, China Jiliang University, 258 Xueyuan Road, Qiantang District, Hangzhou 310018, China; 3 Jiangxi Cash Crops Research Institute, 4 Fuzhou Branch Road, Donghu District, Nanchang 330203, China; 4 College of Agriculture, Tongren Polytechnic College, 2 Ziyou Road, Bijiang District, Tongren 554300, China

**Keywords:** light trap, attract and kill, *Empoasca onukii*, trap design, escape prevention

## Abstract

Device structure, light source height, and climatic factors can potentially affect the catching of target pests in light traps. In this study, the installation of an anti-escape cover in a newly designed light trap significantly increased the number of catches of tea leafhoppers, *Empoasca onukii*, an economically significant pest of tea gardens, and it prevented 97.95% of leafhoppers from escaping. A series of assessments were performed in the field and showed that the optimal trapping window of the light trap was between 1.5 and 2.5 h (2 ± 0.35 h) after sunset, and the starting time of the window was positively correlated with the sunset time. The number of leafhopper catches decreased sharply when the height of the light source was above the flight height range of *E. onukii* adults. The height of the light source was optimal between 20 and 40 cm above the tea canopy. The efficacy of the light traps for capturing leafhoppers decreased in the autumn peak period. High numbers of leafhopper catches by the newly designed light trap in the summer could reduce *E. onukii* population sizes in the autumn. Overall, the newly designed light trap can be used to reduce *E. onukii* adult populations in tea gardens.

## 1. Introduction

Light traps are commonly used to trap and monitor pests; these traps take advantage of the positive phototaxis behavior of insects [1,2]. When airborne insects dash against (e.g., beetles) [3] or gather near (e.g., mosquitoes) [4] artificial light sources, the light source can be combined with other devices (water basin, electric grid, or suction fans) to “attract and kill” these insects [5].

The target tea pests of light traps include the tea leafhopper *Empoasca onukii* and the geometrid moth *Ectropis grisescens*, which are common and economically significant pests in Chinese tea gardens [6,7]. In a previous study, we designed a light trap for these two tea pests [5]. A light-emitting diode (LED) was used as the light source, which emits narrow wavelengths to attract target pests and reduces the number of catches of non-target insects, and the downdraft airflow created by a rotary fan was used to increase the mortality of small-bodied pests. The area of organic tea gardens is continually increasing. Geometrid pests can be controlled using synthetic sex pheromones [8] or viruses [9]. Chemical pesticides remain the main method used for the control of *E. onukii*, and light traps require improvement for them to be used for tea leafhopper control on a large scale [10]. *E. onukii* nymphs and adults suck the sap of tea shoots and leaves with their piercing–sucking mouthparts, and adult females lay their eggs on tea shoots and tender leaves, which stunts the growth of the apical shoots and leads to a decrease in tea production and quality [6]. In most tea gardens, tea leafhoppers have 9–17 generations per year. The mean development times for eggs and nymphs are 8.0 and 14.1 d at a constant 25 °C and 5.8 and 8.4 d at 31 °C. The adult life span of most leafhoppers is 14–21 d [6,11].

There are several challenges associated with the use of light traps for controlling tea leafhoppers. First, tea leafhoppers show a negative geotaxis in closed spaces without a host and tend to crawl upward and look for cracks. The entrances to the containers of common light traps in tea gardens are open, given that these traps lack anti-escape doors or covers; thus, some of the tea leafhoppers trapped in light traps can escape from the containers when the fan stops working. This defect in the design of these traps has long been overlooked. Thus, escaping leafhoppers congregating around traps might increase the density of the pest population. Second, the height of the light source of the light trap in some tea gardens can exceed the flight height range of tea leafhoppers, which reduces the number of leafhoppers captured by the light trap. Third, electricity can be rapidly exhausted by light traps that work for long periods at night in a continuously rainy climate, which can cause the light trap to cease functioning during critical periods.

Structural improvements can enhance the efficacy of light traps [12]. However, observations of how leafhoppers escape from light traps are limited. Previously, we have found that the height of the light source can affect the number of *E. onukii* catches by light traps [5]. The height of the tea canopy constantly changes, especially after pruning; this can eventually cause the height of the light source to exceed the flight height range of *E. onukii*, which may lead to a sharp decline in the trapping efficacy of the light traps. In addition, the optimal range of the light source height remains unclear. The flight activity of many insects often occurs over a narrow time window, and the trapping and killing efficacy of the light traps are maximized during this key period [2]. Color sticky traps are commonly used to assess the flight activity of insects [1]. We found that the peak flight times of *E. onukii* were at dawn and dusk using color sticky cards [13]. However, the optimal trapping window for the use of light traps for catching *E. onukii*, including whether the optimal trapping window for light traps and color sticky cards is the same, remains unclear. In theory, the working time of the light trap only needs to cover the peak flight period of *E. onukii*, given that this would both save energy and reduce the number of catches of non-target insects.

Ensuring that plant protection devices are effective, environmentally friendly, and energy-efficient is critical [14]. In this study, we designed a new light trap for *E. onukii*. In contrast to the original light trap [5], the new light trap only uses a single spectrum of blue light to attract *E. onukii* adults; it also features updraft airflow and an anti-escape cover in the pest container, which significantly increases the number of catches of *E. onukii* adults and reduces accidental catches of natural enemies. We also studied the effects of the light source height and the trapping window on the number of catches of *E. onukii* adults to determine the optimal height range of the light source and working time of the light trap for attracting *E. onukii* adults. We also carried out field experiments to evaluate the ability of our new light trap to effectively control *E. onukii* populations in different tea gardens. The aim of this study was to determine the efficacy of the new light trap for controlling *E. onukii* populations and provide valuable information that will aid the development of leafhopper light-trapping technology.

## 2. Results

### 2.1. Improvements in Device Structure Increase Leafhopper Catches

**Figure 1:**
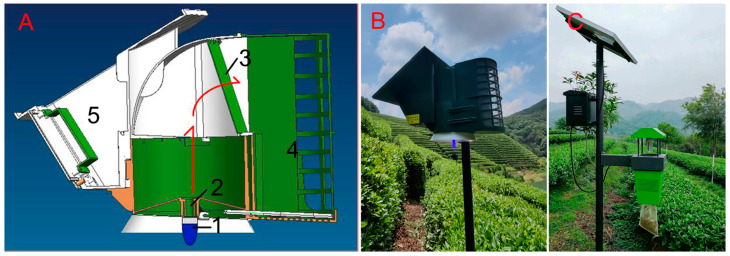
Light traps used to attract and kill *Empoasca onukii* in tea gardens. (**A**) The internal structure of the newly designed light trap (light trap 1); insects attracted to the LED light source (1, 420 nm, 1 W) are sucked by the updraft air flow generated by the rotary fan (2) into the holding container (4) above the lamp. When the light trap stops working, the anti-escape cover (3) automatically closes to prevent the escape of leafhoppers. The light trap operates using solar energy, and the battery and circuit board are placed in the control box (5) of light trap 1. (**B**) Photograph of the new light trap 1. (**C**) Photograph of the positive control light trap (light trap 3). Insects attracted to the LED light source (385 and 420 nm, ratio 1:1, 8 W) are sucked by the downdraft air flow into the holding container beneath the lamp.

The power and spectral range of the light source in the newly designed light trap (light trap 1, Figure 1A,B) were reduced relative to the control light trap (light trap 3, Figure 1C). The installation of the anti-escape cover increased the number of leafhopper catches.

In the summer activity peak of *E. onukii*, the leafhopper relative population density (LRPD) was 52.2 ± 9.55; the main pests trapped in the light trap included Cicadellidae leafhoppers, Geometridae and Olethreutidae moths, and Cecidomyiidae and Simuliidae pests; other insects captured included staphylinid beetles and alate ants (Table 1, Figure S1A). The number of catches of *E. onukii* adults was significantly higher in the newly designed light trap (light trap 1, 1152.33 ± 127.49) than in the other two light traps (F = 162.14, *df*_1_ = 2, *df*_2_ = 6, *p* < 0.001). The number of *E. onukii* adults in light trap 2 without the anti-escape cover was the lowest (only 2.05% of that in light trap 1), indicating that most of the trapped leafhoppers escaped from the holding container of light trap 2. Geometrid moths are major pests of tea gardens, and several were captured in light trap 3; there was no significant difference in the number of Geometridae moths in light trap 1 and light trap 2, indicating that the presence or absence of an anti-escape cover had no effect on the trapping efficacy of Geometridae moths. The number of catches of Lepidoptera pests and Diptera pests was highest in light trap 1 and light trap 3, and no significant differences in the number of captures of these pests were observed between them. The total catches of staphylinid beetles, ants, and other insects were highest in light trap 3 (F = 115.17, *df*_1_ = 2, *df*_2_ = 6, *p* < 0.001).

In the autumn activity peak of *E. onukii*, the LRPD was 37.6 ± 6.50; the main pests trapped in the light trap included Cicadellidae leafhoppers, Geometridae moths, and Cecidomyiidae and Simuliidae pests; other insects included lady beetles and alate ants (Table 1, Figure S1B). The number of catches of *E. onukii* adults was significantly higher in light trap 1 than in the other two light traps (F = 21.254, *df*_1_ = 2, *df*_2_ = 6, *p* = 0.002). The number of *E. onukii* catches was lowest in light trap 2 without the anti-escape cover (only 17.8% of that in light trap 1). The number of catches of gall mosquitoes increased and exceeded 4000 in a single night in light trap 1 (Figure S1B). The number of catches of Lepidoptera pests and Diptera pests was highest in light trap 3. The total number of catches of lady beetles, ants, and other insects was highest in light trap 3 (F = 16.87, *df*_1_ = 2, *df*_2_ = 6, *p* < 0.003), which indicated that increases in the light source spectrum and power led to increases in the number of catches of natural enemies.

### 2.2. Trapping Window of Leafhoppers at Dusk

The optimal trapping window of the light trap was within 1.5 to 2.5 h (2 ± 0.35 h, Table 2) after sunset, which was significantly longer than that of the yellow sticky cards (0.8 ± 0.27 h, *t* = 6.0, *df* = 4, *p* = 0.004). From the end of July to mid-September, the starting time of the trapping window changed from 6:30 to 5:30.

**Figure 2:**
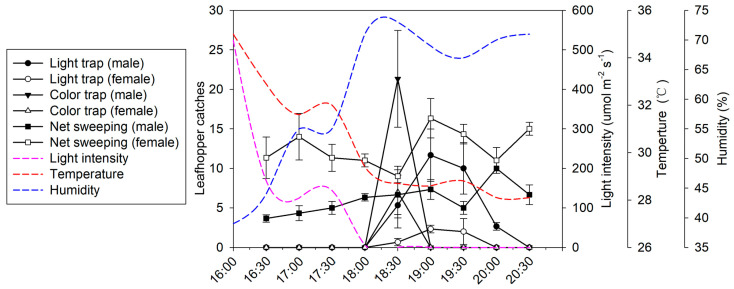
The trapping window of *Empoasca onukii* adults at dusk by the light trap and the yellow sticky card traps in the tea garden. The light trap (circle), yellow sticky cards (square), and sweeping net (triangle) were used to capture *E. onukii* adults every half hour from 16:00 to 20:30 in the tea garden on 31 August 2019. The climatic factors, including light intensity (red dashed line), temperature (blue dashed line), and humidity (pink dashed line), varied from the afternoon to the evening. Climate data were obtained from the meteorological station in the tea garden.

The temperature and light intensity at dusk in the tea garden gradually decreased, and the humidity gradually increased (Figure 2 and Figure S2). From the end of July to mid-September, the period of visible light decreased, and the sun set earlier (Table 2). There was a significant positive correlation between the starting time (*t*_1_) of the trapping window and the sunset time (*t*_s_) for both light traps (correlation coefficient *r* = 0.904, *p* = 0.035) and yellow sticky cards (*r* = 0.969, *p* = 0.006). The light intensity at the starting time of the trapping window was low, and the maximum was 58 μmol·m^−2^·s^−1^.

During the experiment, the number of catches of male *E. onukii* in the light trap was significantly higher than the number of catches of females (*p* < 0.001, Table 2). With the exception of September 14 (*t* = 1.29, *df* = 17, *p* = 0.253), the number of male catches in the yellow sticky cards was significantly higher than the number of female catches (*p* < 0.05). However, the number of female catches from net sweeping was significantly higher than the number of male catches (*p* < 0.001, Table 2), indicating that the number of airborne male *E. onukii* adults was much higher than that of airborne females at dusk and that the duration of male flight activity was also longer than that of females.

The trapping window was the period during which the yellow sticky cards or the light traps were active. The starting time of the trapping window was *t*_1_, and the ending time was *t*_2_. *t*_s_ is the theoretical sunset time. The number of leafhoppers was the number of catches (mean ± SD) by light trap 3, yellow sticky cards, and net sweeping every half hour. The significance of differences in the number of male and female leafhopper catches was analyzed using a paired *t*-test (*p* < 0.05).

### 2.3. The Number of Leafhopper Catches Sharply Declined When the Height of the Light Source Exceeded Their Flight Height Range

**Figure 3:**
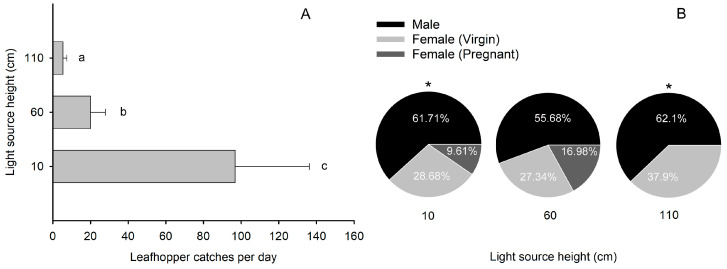
Catches of *Empoasca onukii* adults in the newly designed light trap under the light source at different heights. (**A**) Number of catches of leafhoppers in the trap with a vertical distance between the light source and tea canopy of 10, 60, and 110 cm. (**B**) Percentage of catches of male, virgin, and pregnant female leafhoppers in the light trap at the three light source heights. Mean values with different letters indicate significant differences within a histogram (*p* < 0.05, one-way ANOVA). * indicates a significant difference between number of male and female catches (*p* < 0.05, paired *t*-test).

The height of the light source had a significant effect on the number of catches of *E. onukii* adults (Figure 3A, *F* = 51.5, *df*_1_ = 2, *df*_2_ = 12, *p* < 0.001). As the height of the light source increased, the number of catches of males (*F* = 62.9, *df*_1_ = 2, *df*_2_ = 6, *p* < 0.001), total females (*F* = 40.2, *df*_1_ = 2, *df*_2_ = 6, *p* < 0.001), and virgin females (*F* = 40.3, *df*_1_ = 2, *df*_2_ = 6, *p* < 0.001) decreased significantly (Figure 3B). There was no significant difference in the number of pregnant female catches at a light source height of 60 cm and 10 cm (*t* = 1.38, *df* = 2.1, *p* = 0.3), and no pregnant female was captured at a light source height of 110 cm. At a light source height of 10 cm (*t* = 8.3, *df* = 2, *p* = 0.014) and 110 cm (*t* = 5.0, *df* = 2, *p* = 0.038), the number of male catches was significantly greater than the number of female catches. However, there was no significant difference between the number of male and female catches in the light trap at a light source height of 60 cm (*t* = 0.87, *df* = 2, *p* = 0.48). The number of virgin females in the light trap was significantly higher than that of pregnant females at all heights tested (*p* < 0.01).

### 2.4. Efficacy of the Newly Designed Light Trap on E. onukii Control

**Figure 4:**
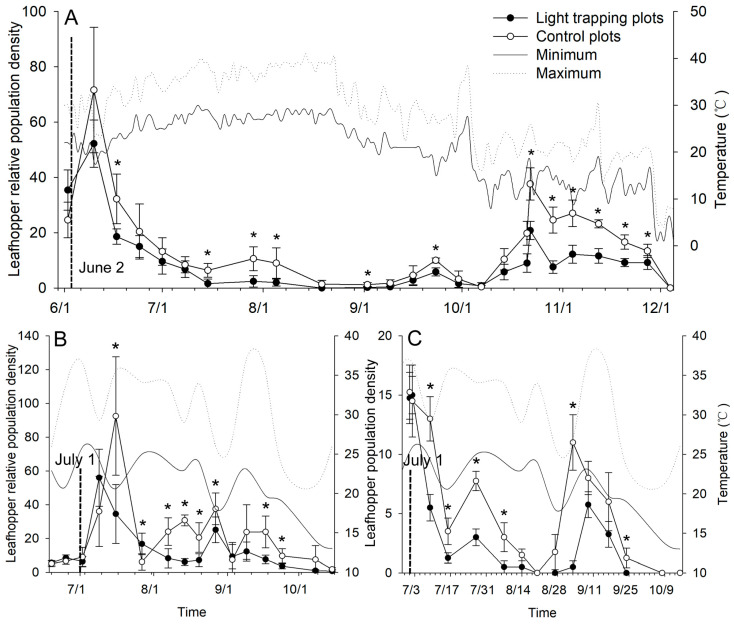
Changes in tea leafhopper population density in the light-trapping plots and control plots. (**A**) Changes in tea leafhopper relative population density (LRPD) in tea gardens in Hangzhou in 2022. (**B**) LRPD dynamics in tea gardens in Tongren in 2023. (**C**) Population dynamics of *Empoasca onukii* in tea gardens in Tongren in 2023. Newly designed light traps were installed in the light-trapping plots, and light traps were absent from the control plot. The solid and dashed lines show changes in the highest and lowest temperature in the tea garden, respectively. The vertical dotted line on the horizontal axis corresponds to the period during which the light traps were active. * indicates significant differences in the mean LRPD or the *E. onukii* population between the light-trapping plots and control plots (simple effect test with Bonferroni’s correction, *p* < 0.05).

In the test conducted in Hangzhou in 2022 (Figure 4A), the mean LRPD in the treatment plots was higher than that in the control plots before the light traps were activated (1 June), and then it was significantly impacted after the light trap worked over time (*p* < 0.05, repeated measures ANOVA, Table S1). The first peak in flight activity occurred on June 10, and the LRPDs in the treatment plots were lower than those in the control (*p* = 0.148). The LRPDs declined and had recovered by July 29, and the LRPDs were significantly lower in the treatment plots than in the control (*p* = 0.009). The second peak occurred on October 22; the LRPDs were still lower in the treatment plots than in the control plots (*p* = 0.001). The number of leafhopper catches in light trap 1 was lower on October 24 than on June 12 (Table 1, *t* = 15.09, *df* = 4, *p* < 0.001), which indicated that (1) the light trap lost its trapping efficacy for leafhoppers in the autumn peak period and (2) a high number of leafhopper catches in summer might inhibit the population of leafhoppers in autumn. The mean LRPD was also significantly affected by the light trap in the test conducted in Tongren in 2023 (*p* < 0.05, repeated measures ANOVA, Figure 4B, Table S1); the first peak in flight activity occurred on 16 July 2023, and the LRPD in the treatment plots was significantly lower than that in the control (*p* = 0.002). In the peaks that occurred on August 14 (*p* < 0.001) and 27 (*p* = 0.017), the LRPDs were lower in the treatment plots than in the control plots. The LRPDs were still lower in the treatment plots than in the control plots on September 17 (*p* = 0.001). After the light traps were activated (1 July), the population density of *E. onukii* declined (July 9; *p* = 0.001) and recovered by July 27 (*p* < 0.001) and September 3 (*p* < 0.001), and the populations were significantly lower in the treatment plots than in the control (*p* < 0.05, repeated measures ANOVA, Figure 4C, Table S1).

**Figure 5:**
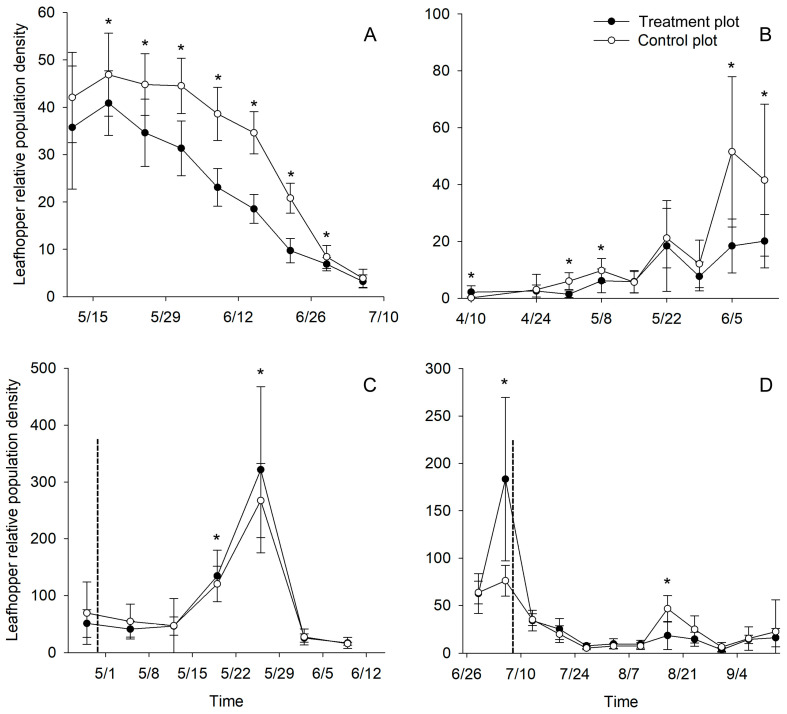
Changes in tea leafhopper relative population density (LRPD) in the light-trapping plots and control plots during the summer occurrence period of *Empoasca onukii* in different tea gardens in 2023. The newly designed light traps were turned on before the summer activity peak of *E. onukii* (LRPD < 5) in tea gardens in Qionglai (**A**) and Wuyishan (**B**) and during the summer activity peak (LRPD > 30) in Songyang (**C**) and Nanchang (**D**). The vertical dotted line on the horizontal axis corresponds to the period during which the light traps were active. * indicates significant differences in LRPD between the treatment plot and control plot (simple effect test with Bonferroni’s correction, *p* < 0.05).

The mean LRPD was significantly affected by the light trap over time in the summer experiments (*p* < 0.05, repeated measures ANOVA, Figure 5, Table S1). In the Qionglai and Wuyishan tea gardens, the light traps were activated before the summer activity peak of *E. onukii* adults. The number of leafhopper catches in the Qionglai tea garden peaked on May 18, and the LRPDs were significantly lower in the treatment plots than in the control plots (*p* = 0.042; Figure 5A). In the Wuyishan tea garden (Figure 5B), the LRPDs were significantly lower in the treatment plots than in the control plots during the summer activity peak on June 5 (*p* < 0.001). In the Songyang and Nanchang tea gardens, the light traps were activated during the peak summer flight period of *E. onukii* adults. In the Songyang tea garden (Figure 5C), the LRPDs were lower in the treatment plots than in the control plots before light traps were activated (April 28), but this difference was not significant (*p* = 0.183). *E. onukii* flight activity peaked on May 26, and the number of catches in the trap was low because the height of the light source was not optimal; consequently, the LRPDs were significantly higher in the treatment plots than in the control plots (*p* = 0.001). In the Nanchang tea garden (Figure 5D), the LRPDs were 2.41 times higher in the treatment plots than in the control before the light traps were activated (July 6, *p* = 0.013); the leafhopper population began to decline and peaked again on August 17. At this time, the LRPDs were significantly lower in the treatment plots than in the control plots (*p* = 0.002), indicating that the light trap had a significant inhibitory effect on the growth of the adult *E. onukii* population.

## 3. Discussion

Ensuring that plant protection devices are effective, energy-efficient, and environmentally friendly is a major goal of current research [14]. We developed a new light trap that is more effective at capturing the target pest than other previously developed light traps; the number of captures of non-target insects by our new light trap was also lower than that by other light traps. We identified the optimal period for employing the light trap, and the power of the light source in our light trap is lower than that of other light traps.

### 3.1. Improved Trapping Efficacy

Although the power of the light source is reduced in the newly designed light trap, the structural improvements to the trap greatly increased the number of adult *E. onukii* catches. This might be explained by the installation of the rotary fan and holding container above the lamp. In most light traps designed to trap pests in tea gardens, the rotary fan is located below the light source in the light trap, which creates an area free of radiation below the trap. In 1972, Wilton and Fay designed a light trap for trapping mosquitoes; they showed that an updraft airflow did not affect the trapping efficacy of small insects and that it could reduce the number of captures of non-target insects [15]. Furthermore, the holding container in our device was fitted with an anti-escape cover. On the non-host plant, *E. onukii* is continuously active, and it shows a negative geotaxis. If the entrance of the holding container (facing up) does not have an anti-escape cover, once the leafhopper adapts to the high-light environment, it can easily escape from the container. We speculated that many of the trapped leafhoppers had escaped from the container without the anti-escape cover, which was the cause of the low efficacy of the light trap for capturing *E. onukii*. Males of the leafhopper can mate multiple times [16]; thus, ensuring the mortality of the trapped leafhoppers is important for ensuring the high efficacy of light traps.

Although the light trapping significantly reduced the number of leafhoppers in the tea garden, there is still a major gap in the control efficacy between light traps and chemical pesticides. There is thus a need to further improve the control efficacy of light traps for *E. onukii*. The number of catches of male leafhoppers is significantly higher than the number of captures of females [17], and the control efficacy of the “attract and kill” strategy depends on whether enough males can be quickly killed over a short period [18]. Active pests (e.g., in flight) are the targets of trapping approaches [19]. We found that *E. onukii* adults on the host exhibited light avoidance responses instead of positive phototaxis when illuminated (unpublished data). Therefore, the key to increasing the number of insect catches in light traps is to increase the flight activity of the target pests. External acoustic noise has been shown to increase the flight activity of the grape leafhopper, *Scaphoideus titanus*, as well as the number of catches of *S. titanus* on sticky traps [20]. Both *E. onukii* and *S. titanus* adults rely on vibrational signals for mating communication. Male leafhoppers are highly active during the mating process. Any external factors that interrupt this process can induce males to take flight [16,21]. The broadcasting of specific noises for disrupting the mating behavior of *E. onukii* in tea gardens can reduce the oviposition rate of *E. onukii* and increase flight activity, which may further increase the number of *E. onukii* adults captured in our new light trap.

The use of multiple attractive cues can improve the efficacy of traps in the field. For example, the brown marmorated stink bug, *Halyomorpha halys*, is an invasive species that has become a major agricultural pest in its invaded range. Vibration signals have been used to increase the efficacy of pheromone traps for this species in light of previous studies of its mating behavior [22]. Plant volatiles can increase the number of adult *E. onukii* captures by color sticky traps in tea gardens [23]. The combination of light and sex pheromones is optimal for attractiing the leopard mothes, *Zetizera pyrina*, in olive orchards [24]. However, the addition of attractive volatiles or vibrational cues may not have a synergistic effect on the new light trap in this study because of the airflow and vibration noise generated by the working fan. The use of additional attractive cues might further enhance the efficacy of traps in which the transmission of attractive cues cannot be compromised by features of the trap design, such as color traps and light traps combined with electrical grids or water basins.

### 3.2. Factors Affecting the Trapping Efficacy of the Light Trap

The height of the trap affects the number of catches of the target pest; this is related to the flight height of the pest under natural conditions [25]. The flight height of the pest thus determines the optimal height of the trap. The flight height varies among insects, and the number of catches by light traps is reduced if the height of the trap exceeds the flight height of the target pest; this has been demonstrated in phlebotomine sand flies [26]. For some insects, such as *Culicoides* midges [27] and *Paederus* beetles [28], a light trap below their main flight height range can also result in a decrease in the number of catches. The height of the trap might not have a significant effect on the number of catches of some insects, such as host-seeking anopheline mosquitoes [29,30]. In a previous study, we found that the height of the light source affected the number of catches of *E. onukii* adults [31]. Wang et al. monitored the flight height of *E. onukii* in tea gardens with color sticky cards and found that the flight height of *E. onukii* varied among seasons [32]. The flight height of *E. onukii* ranged from 40 cm below to 60 cm above the tea canopy. In spring (May) and autumn (October), the flight height of *E. onukii* was mainly concentrated under the tea canopy. In early summer (June), individuals were mainly observed flying 20 cm above the tea canopy. In the summer, when the temperature is higher (July), individuals were mainly observed flying between 40 and 60 cm above the tea canopy. Therefore, the height of the light source was optimal between 20 and 40 cm above the tea canopy. If the height of the light source is lower than this range, the light source is blocked by the growing tea branches in spring and summer. Placing the light trap at heights above 60 cm would lead to a sharp decrease in the number of catches.

The flight activity of leafhoppers also affects the effectiveness of the light trap [33]. Although the LRPDs in the treated plots and control plots were similar, the number of catches of *E. onukii* adults in the light trap during the two activity peaks (summer and autumn) significantly differed (Table 1). That is, the light trap lost its trapping efficacy for leafhoppers during the autumn peak; this same pattern was also observed in a trapping study of the maize leafhopper, *Cicadulina bipunctata* [34]. Climatic factors (e.g., moon phase, cloud cover, wind speed, temperature, and humidity) have significant effects on the number of catches of leafhoppers in light traps [35]. Temperature is probably the most important climatic factor, given that the flight activity of many insects is affected by the ambient temperature. For example, the trapping efficacy of male *Lymantria monachal* is highest when the night temperature is between 15 and 22 °C. However, when the temperature is below 10 °C at night, the traps are not effective because the males are not active [36]. The flight activity of many leafhoppers, such as *Graminella nigrifrons* [37], *Nephotettix virescens*, and *Nephotettix nigropictus* [38], is significantly reduced at low temperatures. The leafhopper *Graphocephala atropunctata* is not observed inside traps unless the night temperature is above 14.5 °C [39]. Sticky card traps can capture insects during the daytime, when the temperature is relatively high; overwintering *E. onukii* adults have been found on these traps in late autumn (November) [34,40]. Therefore, sticky card traps are more suitable for monitoring changes in the numbers of *E. onukii* adults in tea gardens compared with light traps. Although determining the treatment thresholds for the effective use of sticky card traps to monitor changes in leafhopper adult populations remains a major challenge [33], identifying the optimal application window of some pest control technologies (e.g., ensuring that they are used during high-activity periods, such as mating) is important for enhancing their efficacy [14].

In contrast to pheromone traps, which mainly capture males, light traps can capture both sexes, and the proportion of males captured was significantly higher than the proportion of females captured. The females captured were likely searching for food or oviposition sites. *E. onukii* is active from spring to autumn and has several generations a year [11]. Because the trapping efficacy of light traps for *E. onukii* adults decreases in autumn, light traps should be used from spring (when *E. onukii* adults first appear in tea gardens) to early autumn. Because the trapping window of color sticky cards is narrow and the trapping peak is earlier than that for light traps (Figure 2 and Figure S2), the effect of temperature on the flight activity of *E. onukii* adults needs to be studied in detail in the laboratory to clarify the ambient temperature threshold below which the light trap should be deactivated in autumn.

The flight activity period varies among nocturnal insects [2]. Accidental catches of non-target insects can be reduced by optimizing the working hours of the light traps. The diel flight activity of *E. onukii* adults was greatest at dawn and dusk and most concentrated within a 2 h period after sunset. Most light traps are controlled by light intensity sensors; thus, a working time of 2 h that covers the flight window of the target pest is generally sufficient.

## 4. Materials and Methods

### 4.1. Light Traps

The newly designed light trap (light trap 1, Figure 1A,B) and its light source were manufactured by Yeehar Agricultural Technology Co., Ltd. (Hangzhou, China). The light source was a blue (420 nm) LED conical lamp (1 W), the wavelength that is most attractive to *E. onukii* leafhoppers [5]. The holding container above the light source collects insects attracted by the light source through the updraft airflow generated by the electric fan. After the fan stops working, the anti-escape cover of the container automatically falls and fits around insects via the action of gravity to prevent leafhoppers from escaping. The wind speed of the inlet (5 cm below the fan, Figure 1A) and outlet (container entry) was 2.03 ± 0.12 m/s and 5.97 ± 0.21 m/s, respectively; the wind speed was measured using an environmental measuring instrument with a 6533-2G sensor (Model 65Ser, Kanomax Inc., Osaka, Japan). The aperture of the peripheral mesh of the holding container was 0.425 mm.

The new light trap without an anti-escape cover was used as a control light trap (light trap 2). A previous light trap [5] designed for the two tea pests was used as a positive control trap (light trap 3), which was manufactured by Zhejiang Top Instrument Co., Ltd., Hangzhou, China. Two types of LED chips (385 and 420 nm, ratio 1:1) were used in the light source (8 W) of light trap 3, and a rotary fan was used to move attracted insects down into the holding container suspended beneath the lamp (Figure 1C). The peripheral mesh of the holding container was 0.425 mm.

### 4.2. Device Structure

Some of the improvements of light trap 1 compared with light trap 3 included the spectrum range and power of the light source, the position of the holding container, and the anti-escape cover. The effect of the anti-escape cover was evaluated by comparing light trap 1 with light trap 2.

Tests comparing insect catches in light traps 1, 2, and 3 were conducted in the peak summer and autumn periods (10 June and 22 October 2022) of *E. onukii* in the organic tea garden located at Hangzhou, Zhejiang Province, China (30.18° N, 120.09° E). Before tests, we used yellow sticky cards to measure the leafhopper relative population density (LRPD) of *E. onukii* adults in the test area of the tea garden [23]. Five yellow sticky cards were installed evenly, 20 cm above the tea canopy in each area for 24 h. The mean number of leafhoppers on the five cards was used as the LRPD of each test area. Three types of light traps were installed in areas where the LRPD was between 40 and 60, and the spacing between each trap was between 50 and 55 m. The vertical height between the light source and the tea canopy was 20 cm. The working time of the light traps was from 15:00 to 22:00, and the catches of dominant tea pests and natural enemies in the holding container were counted the following day. The tea pests included Cicadellidae leafhoppers, Lepidoptera moths, and Diptera pests. The natural enemies included Staphylinidae and Coccinellidae beetles, Hymenoptera parasitic wasps and ants, and Chrysopidae lacewing. Trials of each type of light trap were repeated three times.

### 4.3. Trapping Window

Tests were conducted to investigate the trapping window of the light trap on the number of catches of *E. onukii* adults from the afternoon to evening during the flight period of *E. onukii*.

Six plots (10 × 10 m) were established with a distance of 50 m between each plot in an organic tea garden in Shaoxing, Zhejiang Province, China (32.57° N, 130.50° E). Light trap 3, yellow sticky cards, and a sweeping net were used to capture *E. onukii* adults every half hour from 16:00 to 20:30 in the tea garden. The leafhoppers in three plots were captured using light traps, and the leafhoppers in the other three plots were captured using yellow sticky cards and a sweeping net. One light trap was installed with the light source 30 cm above the tea canopy in each of the three plots. The working time of the traps was from 16:00 to 20:30, each container was replaced with a new one every half hour, and the number of leafhoppers inside was counted. Three yellow sticky cards were installed evenly 10 cm above the tea canopy in each of the other three plots; they were replaced every half hour, and the number of leafhoppers on each card were counted. When each yellow sticky card was being replaced, we swept the tea canopy four times with a sweeping net at a fixed position 10 m away from the card and counted the number of leafhoppers in the net. Climate data were obtained from the meteorological station in the tea garden. The tests were conducted on 28 July; 8, 9 August and 31; and 14 September 2019.

### 4.4. Light Source Height

This experiment was carried out to determine whether a light source positioned high in the canopy (vertical height > 1 m) reduces the efficacy of the light traps for attracting and killing *E. onukii* adults.

Before the tests, we used yellow sticky cards to measure the LRPD in the tea garden in Hangzhou. We then selected the plots (10 × 10 m) where the LRPD was between 10 and 20. The distance between each plot was between 50 and 55 m. We randomly selected three plots and installed one newly designed light trap (light trap 1, Figure 1B) in each plot. The three vertical heights between the light source and the tea canopy tested were 10, 60, and 110 cm. After each test, we randomly selected three plots in the remaining plots and repeated the test. The tests were repeated five times from 8 to 12 June 2022. The light trap automatically turned on after dark and turned off after 3 h. The numbers of male and female leafhoppers (virgin and pregnant) in the trap were determined at 10:00 the following day.

### 4.5. Efficacy of the Newly Designed Light Trap on E. onukii Control

Experiments were carried out to determine whether the newly designed light trap could effectively control *E. onukii* in the field.

*Test 1: Efficacy during the two peak periods.*

The tea garden was divided into five treatment plots and five control plots (10 × 10 m) in an organic tea garden in Hangzhou in 2022. The test was repeated in Tongren, Guizhou Province (27.74° N, 108.91° E), in 2023, and the tea garden was divided into three treatment plots and three control plots (10 × 10 m). The distance between each plot was between 50 and 55 m. One newly designed light trap was installed in each of the five treatment plots, and there was no light trap in the control plot. The height of the light source was regularly adjusted to ensure that it was 10–20 cm away from the tea canopy. Yellow sticky cards were used to measure the LRPD in the treatment and control plots. After the survey of the LRPDs of the plots on June 2 in Hangzhou and July 1 in Tongren, the light traps were turned on and were active from the summer peak to the autumn peak of *E. onukii*, during which the LRPD was measured approximately every 7 days. In the Tongren test, we investigated the population dynamics of *E. onukii* using traditional methods [41]. The central row (1.2 × 10 m) of tea plants in each plot was used as the sample site. On each sample site, 100 tea shoots were randomly selected, and counts of *E. onukii* nymphs and adults on the three leaves below the bud per shoot were made. The population density was measured approximately every 7 days. Temperature data were collected from the meteorological station in the tea garden.

*Test 2: Efficacy during the summer.*

Tests were conducted during the summer in four different organic tea gardens in 2023. Each tea garden was divided into three treatment plots and three control plots (25 × 25 m); the distance between each plot was 100 m to prevent the light traps in the treatment plots from affecting the control plots. Five newly designed light traps were uniformly installed in the treatment plots, and no light trap was installed in the control plots. The light traps were turned on before the summer peak in *E. onukii* activity (LRPD < 5) in the tea gardens in Qionglai, Sichuan Province (30.25° N, 130.27° E) and Wuyishan, Fujian Province (30.43° N, 117.59° E). In the other two tea gardens in Songyang, Zhejiang Province (28.53° N, 119.46° E) and Nanchang, Jiangxi Province (28.38° N, 116.02° E), the light traps were turned on during the summer peak in *E. onukii* activity (LRPD > 30). The height between the light source and the tea canopy was approximately 20 cm, except for the test in Songyang, where the height was 110 cm. Five yellow sticky cards were hung evenly in each plot, and surveys of the LRPD were conducted every 7 days. The control effectiveness of the newly designed light traps against *E. onukii* was evaluated by comparing the LRPDs in the treatment and control plots.

### 4.6. Data Analysis

In experiment 4.2, the number of catches of various insects in the three types of light trap was compared using one-way ANOVA and LSD tests in SPSS 19.0 (IBM Corp., Armonk, NY, USA) to evaluate the effect of the device structure on the ability of the device to attract and kill tea pests and natural enemies.

In experiment 4.3, we collected *E. onukii* adults every half hour with a light trap, yellow sticky cards, and sweeping net. The number of male and female leafhopper catches from the three different methods was compared using a paired *t*-test. The trapping window was defined as the period during which the yellow sticky cards and the light trap captured *E. onukii* leafhoppers. The starting time of the trapping window was *t*_1_, the ending time was *t*_2_ (Table 1), and the duration of the trapping window was *t*_t_ = *t*_2_ – *t*_1_. The *t*_t_ values between the light trap and the yellow sticky cards were compared using a paired *t*-test. Finally, the Pearson correlation coefficients between *t*_1_ and sunset time (*t*_s_) were calculated to evaluate the effect of sunset time on the starting time of the trapping window of the yellow sticky cards and light traps.

In experiment 4.4, one-way ANOVA and LSD tests were used to analyze the significance of differences in the number of catches of *E. onukii* adults in the light traps at different light source heights. All data were log_10_ transformed (*x* + 0.1) to ensure homogeneity of variances. Because no pregnant females at a light source height of 110 cm were captured, an unpaired *t*-test was used to determine whether there was a significant difference in the capture of pregnant females at a height of 10 cm and 60 cm. The difference in the number of catches of males and females was analyzed using a paired *t*-test.

In experiment 4.5, a repeated measures ANOVA was conducted (using time as the within-group factor) to analyze the effects of the light trap on changes in the LRPDs or the population dynamics of *E. onukii*. A simple effect test (Bonferroni’s correction, *p* < 0.05) was then conducted to analyze the differences in the *E. onukii* population in the treatment and control.

## 5. Conclusions

The responses of insects to light are diverse. Therefore, effectively designing a light trap requires a thorough understanding of the behaviors of target insects. On the basis of the phototaxis, negative geotaxis, and flight activity of *E. onukii* adults, the design of the light source and anti-escape device in the new light trap significantly improved the trapping efficacy of *E. onukii* adults. The trapping window of the light trap on *E. onukii* was longer than that of the color sticky card, the trapping peak was later than that of the color sticky card, and the starting time was positively correlated with the sunset time. The optimal height of the light source was concentrated between 20 cm and 40 cm above the tea canopy. However, the trapping efficacy of the light trap on *E. onukii* significantly decreased in autumn. The newly designed light trap in this study can significantly reduce adult *E. onukii* populations in tea gardens; these traps are also low-cost and highly versatile.

## Figures and Tables

**Table 1:** Species and number of catches of insects by three different light traps in a single night.

		Summer					Autumn				
		Trap 1	Trap 2	Trap 3	*F*	*p*	Trap 1	Trap 2	Trap 3	*F*	*p*
Tea pest											
Hemiptera	Cicadellidae	1152.33 ± 127.49 a	23.67 ± 6.43 c	308.67 ± 53.15 b	162.14	<0.001	39.33 ± 8.74 a	7.0 ± 2.0 c	21.0 ± 5.57 b	21.25	0.002
Lepidoptera	Geometridae	32.0 ± 6.08 b	28.67 ± 3.06 b	65.0 ± 8.89 a	28.97	0.001	13.67 ± 4.51 a	13.0 ± 7.21 a	24.67 ± 8.74 a	2.60	0.154
	Olethreutidae	44.33 ± 5.03 b	8.0 ± 3.61 b	19.67 ± 8.14 a	29.59	0.001	0	0	0		
	Others	12.67 ± 3.51 a	10.67 ± 6.23 a	32.67 ± 14.57 b	5.01	0.053	8.33 ± 2.52 b	6.67 ± 4.62 b	34.33 ± 14.05 a	9.63	0.013
	Total	89.0 ± 10.39 a	47.33 ± 4.93 b	117.33 ± 27.47 a	12.59	0.007	22.0 ± 6.25 b	19.67 ± 11.72 b	59.0 ± 20.66 a	7.26	0.025
Diptera	Cecidomyiidae	249.33 ± 61.09 a	218.33 ± 72.22 a	277.67 ± 77.41 a	0.53	0.614	4084.0 ± 651.75 a	2916.67 ± 940.69 a	4560.0 ± 799.02 a	3.30	0.108
	Simuliidae	450.0 ± 53.51 a	23.67 ± 16.62 c	200.0 ± 74.18 b	47.78	<0.001	0	0	0		
	Others	28.0 ± 14.18 a	32.0 ± 15.52 a	58.67 ± 33.83 a	1.58	0.282	57.0 ± 19.29 b	41.67 ± 22.5 b	123.0 ± 39.13 a	6.98	0.027
	Total	727.33 ± 57.84 a	274.0 ± 55.46 b	536.33 ± 172.4 a	12.9	0.007	4141.0 ± 670.84 ab	2958.33 ± 962.53 b	4683.0 ± 837.19 a	3.37	0.104
Natural enemy											
Coleoptera	Staphylinidae	1.0 ± 1.0 b	0.67 ± 0.58 b	6.67 ± 2.08 a	18.06	0.003	0.67 ± 0.58 a	0.67 ± 0.58 a	3.33 ± 2.31 a	3.56	0.096
Hymenoptera	Formicidae	2.33 ± 0.58 b	0 b	11.0 ± 2.31 a	18.51	0.003	0.67 ± 0.58 b	0.33 ± 0.58 b	8.67 ± 2.08 a	40.07	<0.001
	Others	0 b	0 b	1.33 ± 0.58 a	16.0	0.004	0.67 ± 1.15 a	0.33 ± 0.58 a	1.67 ± 0.58 a	2.17	0.196
	Total	3.33 ± 0.58 b	0.67 ± 0.58 b	19.0 ± 2.65 a	115.17	<0.001	2.0 ± 1.0 b	1.33 ± 1.53 b	13.67 ± 6.52 a	16.87	0.003

Trap 1 is the newly designed light trap with updraft airflow and an anti-escape cover, trap 2 is the newly designed light trap without the anti-escape cover, and trap 3 is the down-draught light trap without an anti-escape cover. Mean values ± SD with different letters indicate significant differences in the number of insect catches among light traps (*p* < 0.05, one-way ANOVA).

**Table 2:** Mean ± SD catches of *Empoasca onukii* adults by light traps, yellow sticky cards, and net sweeping from the afternoon to the evening in the tea garden.

Date	28 July		8 August		9 August		31 August		14 September	
*t*_s_	18:56		18:48		18:47		18:36		18:07	
Light trap	*t*_1_	*t*_2_	*t*_1_	*t*_2_	*t*_1_	*t*_2_	*t*_1_	*t*_2_	*t*_1_	*t*_2_
	18:30	20:00	18:00	20:00	18:00	20:00	18:00	20:00	17:30	20:00
Male	12.89 ± 6.29		1.75 ± 1.22		5.17 ± 3.86		7.41 ± 4.72		12.07 ± 10.15	
Female	5.56 ± 4.90		0.17 ± 0.39		1.33 ± 2.02		1.25 ± 1.36		2.73 ± 3.47	
*t*	11.36		5.06		5.90		4.68		4.91	
*p*	<0.001		<0.001		<0.001		<0.001		<0.001	
Sticky card	*t*_1_	*t*_2_	*t*_1_	*t*_2_	*t*_1_	*t*_2_	*t*_1_	*t*_2_	*t*_1_	*t*_2_
	18:30	19:30	18:30	19:00	18:30	19:00	18:00	18:30	17:30	18:30
Male	6.66 ± 5.85		2.33 ± 0.58		11.67 ± 3.51		21.33 ± 7.51		21.5 ± 20.15	
Female	5 ± 6.03		0		0		7.0 ± 4.0		11.0 ± 7.75	
*t*	7.91		7		5.75		7.07		1.29	
*p*	0.001		0.02		0.029		0.019		0.253	
Net sweeping										
Male	4.25 ± 2.74		3.89 ± 1.37		4.15 ± 2.25		6.11 ± 2.17		6.19 ± 2.68	
Female	12.37 ± 4.46		9.48 ± 2.87		10.78 ± 3.04		12.59 ± 2.98		22.74 ± 4.61	
*t*	10.45		8.46		14.42		8.92		19.41	
*p*	<0.001		<0.001		<0.001		<0.001		<0.001	

## Data Availability

Data are contained within the article and Supplementary Materials.

## Supplementary Materials

The following supporting information can be downloaded at: https://www.mdpi.com/article/10.3390/plants13020241/s1, Figure S1: Insects trapped in the newly designed light trap during the summer (A, June 12) and autumn (B, October 24) activity peaks of *Empoasca onukii* in tea gardens in 2022; Figure S2: The trapping window of *Empoasca onukii* adults at dusk by the light trap and the yellow sticky card traps in the tea garden on 28 July, 8 and 9 August, and 14 September 2019; Table S1: Results of the repeated measures ANOVA conducted to analyze changes in tea leafhopper relative population density (LRPD) and the *Empoasca onukii* population in Section 4.5.
