# Supplementary material for: Device Structure, Light Source Height, and Sunset Time Affect the Light-Trap Catching of Tea Leafhoppers
Source: Plants (Basel). 2024 Jan 15;13(2):241. doi: 10.3390/plants13020241 (PMC10820048; doi:10.3390/plants13020241)
Supplement: Supplementary file 1 [file plants-13-00241-s001.zip › plants-2793441-supplementary/table s1.pdf]

Table S1. Results of the repeated measures ANOVA conducted to analyze changes in tea leafhopper relative population density (LRPD) and the *E. onukii* population in section 4.5.

Field location	Mauchly	Time		$T \times L$	
	p	F	p	F	p
Hangzhou	0.00	58.89	***	3.71	*
Tongren	0.00	35.45	***	9.47	***
Tongren[a]	0.00	104.75	***	9.5	***
Qionglai	0.02	162.38	***	3.94	*
Wuyishan	0.00	51.86	***	11.2	**
Songyang	0.01	64.24	***	13.57	**
Nanchang	0.00	39.01	***	8.62	**

F values and significance levels from the repeated measures ANOVA are shown for two main effects and their interactions. * $0.01 \le p < 0.05$, ** $0.001 \le p < 0.01$, *** $p < 0.001$, ns = not significant, $n = 5$. When Mauchly's $p < 0.05$, Greenhouse–Geisser estimates were used to correct for sphericity. T = time, L = light trap.

a Data of the *E. onukii* population from a traditional method.
