# Supplementary figures and images for: Device Structure, Light Source Height, and Sunset Time Affect the Light-Trap Catching of Tea Leafhoppers
Source: Plants (Basel). 2024 Jan 15;13(2):241. doi: 10.3390/plants13020241 (PMC10820048; doi:10.3390/plants13020241)

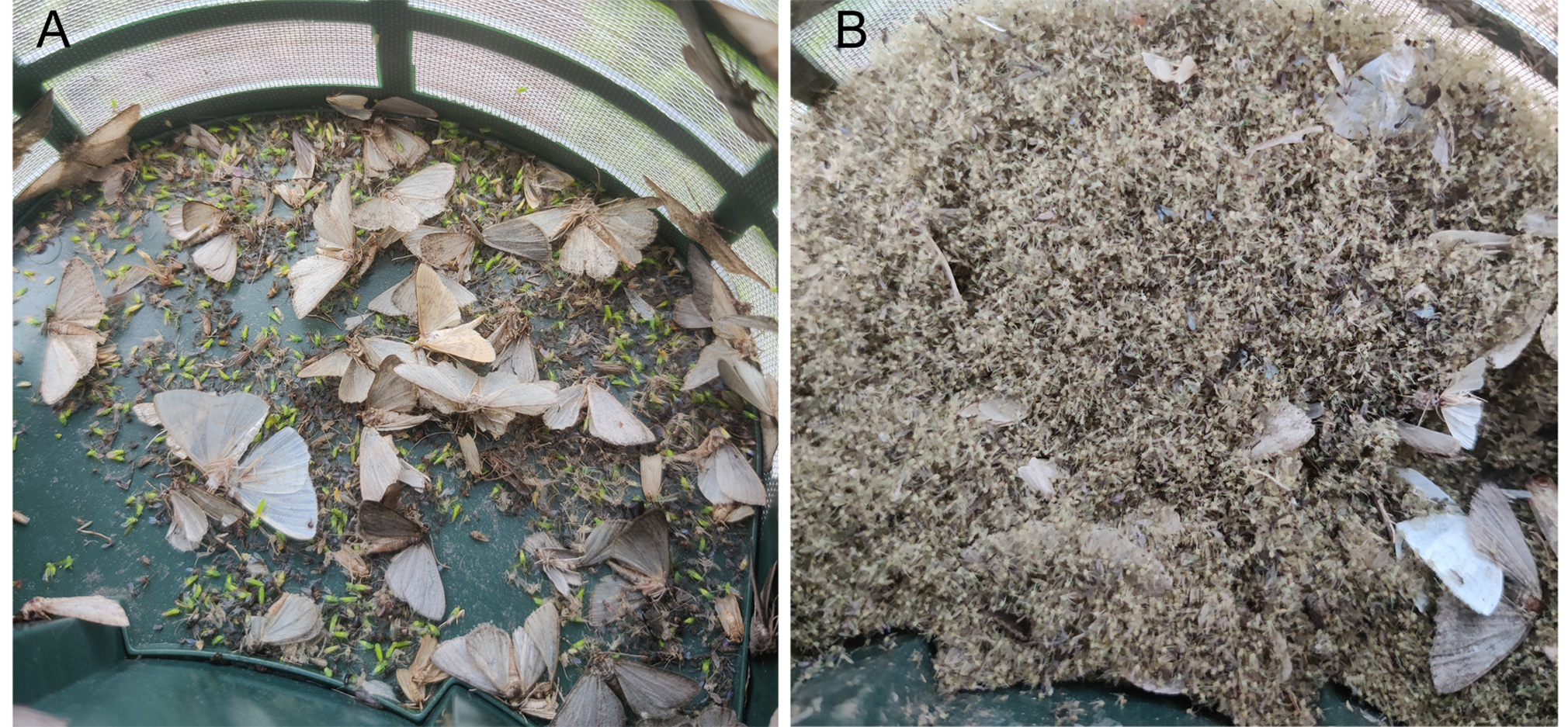

Supplement: Supplementary file 1 [file plants-13-00241-s001.zip › plants-2793441-supplementary/Supplementary Figures/Figure S1.tif]

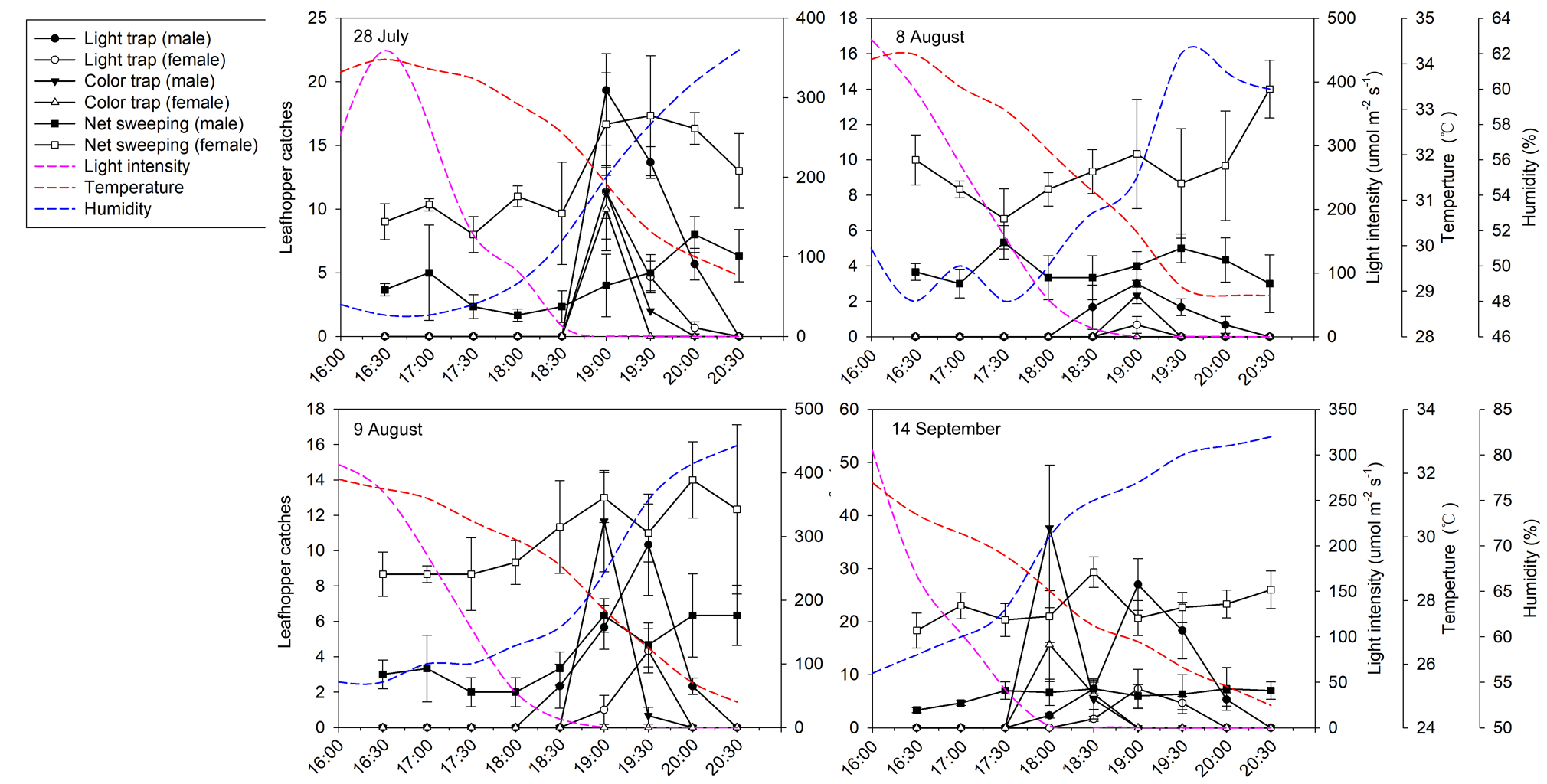

Supplement: Supplementary file 1 [file plants-13-00241-s001.zip › plants-2793441-supplementary/Supplementary Figures/Figure S2.tif]
